# Supplementary material for: mRNA display with library of even-distribution reveals cellular interactors of influenza virus NS1
Source: Nat Commun. 2020 May 15;11:2449. doi: 10.1038/s41467-020-16140-9 (PMC7229031; doi:10.1038/s41467-020-16140-9)
Supplement: Supplementary file 1 — Supplementary Information [file 41467_2020_16140_MOESM1_ESM.pdf]

Figure S1

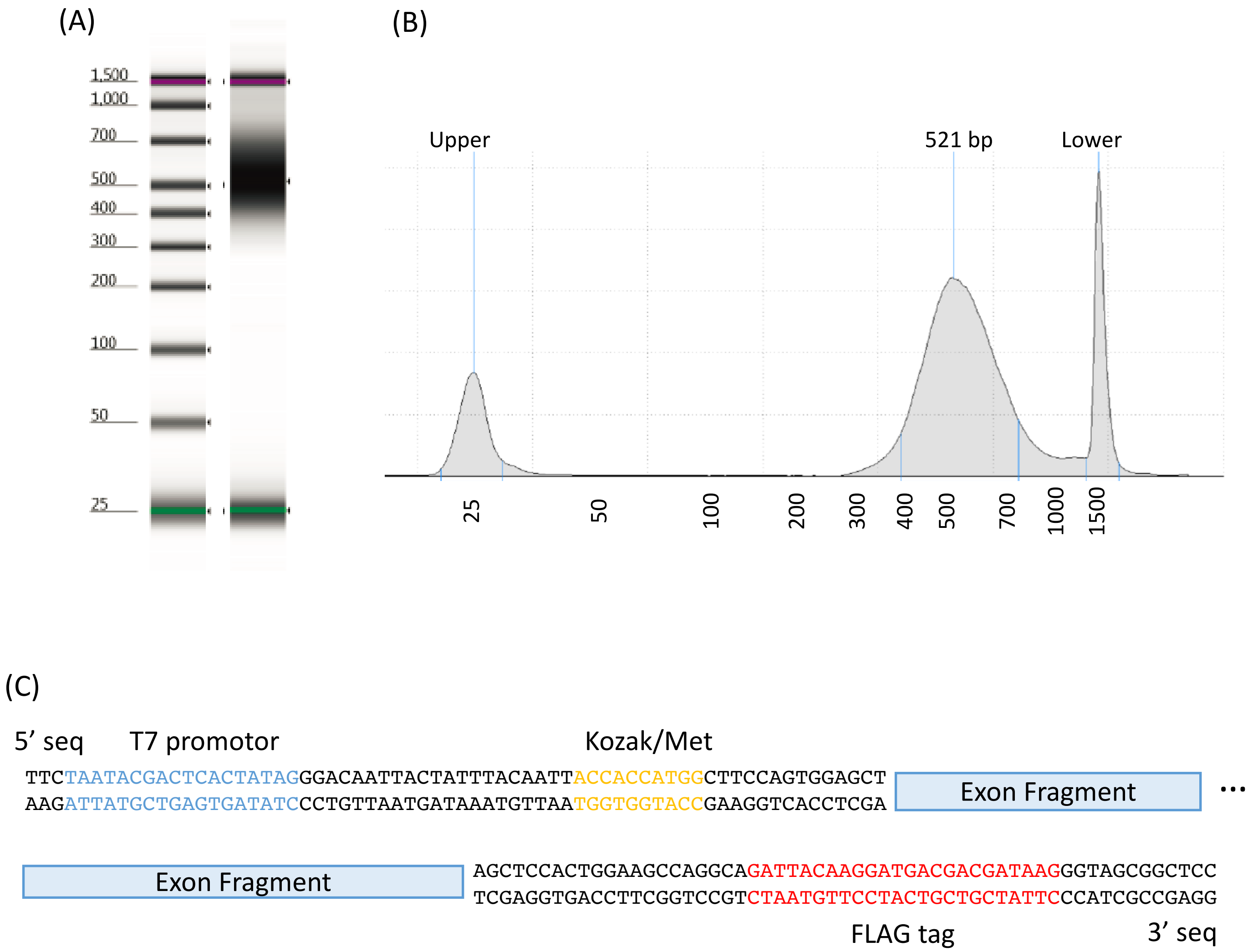

Supplementary Figure 1: Generation of exon library for mRNA display

(A-B) Gel picture (A) and histogram (B) shows the distribution of fragment sizes of the input exon library. Double-strand DNAs, from 25 bp and 1500 bp, were loaded as size markers. (C) Schematic diagram shows the construct of exon library for mRNA display. T7 promotor is colored in blue, Kozak sequence is colored in yellow, and FLAG affinity tag is colored in red. Enriched exon fragment is shown as the light blue box.

Figure S2

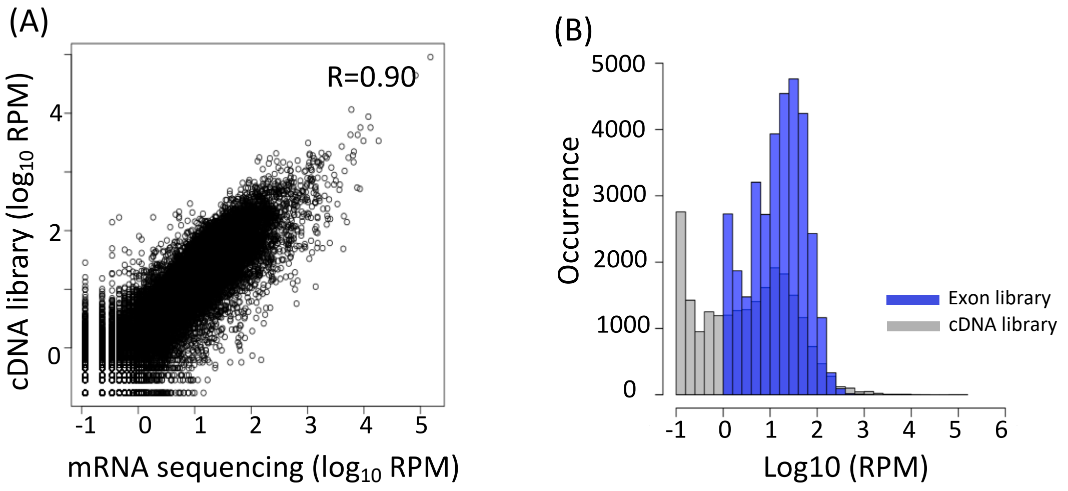

Supplementary Figure 2: Quality control of the exon library

(A) The scatter plot shows the correlation of cDNA library and mRNA sequencing. Dots represent the frequency of a transcript/cDNA in each library. (B) The histogram shows the frequency of genes in exon library and cDNA library counted by HTseq software. Blue bars represent the distribution of exon library, and gray bars correspond to cDNA library.

Figure S3

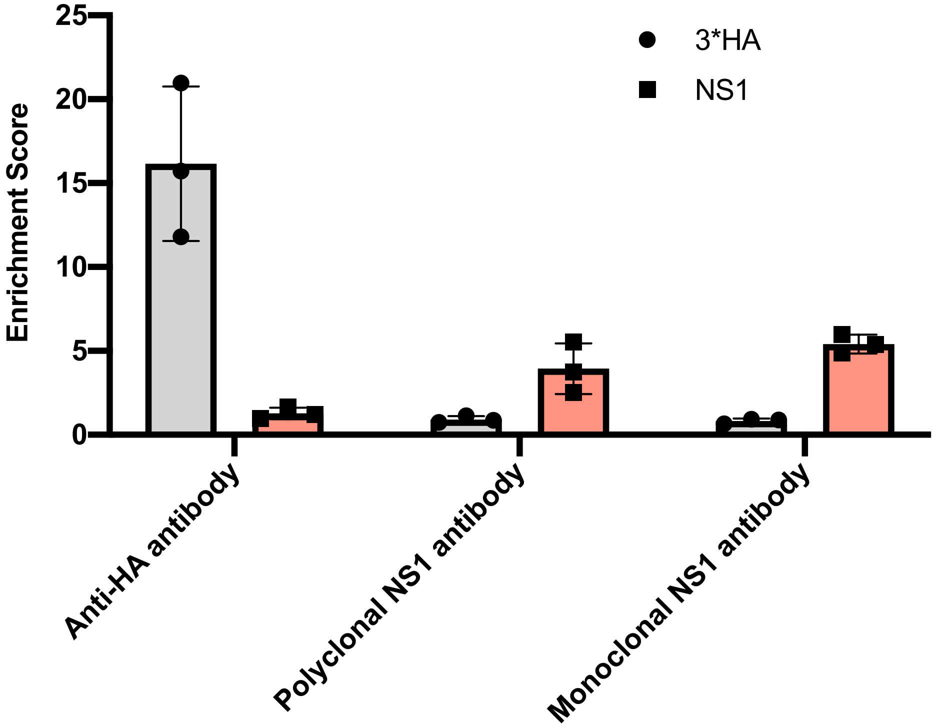

Supplementary Figure 3: Feasibility of detecting protein-protein interaction using exon display
Enrichment of indicated target protein sequences after one round of enrichment. 3*HA tag with linker sequence and influenza NS1 gene were spiked into the human exon library at a frequency of 0.01%. Anti-HA antibodies, monoclonal and polyclonal anti-NS1 antibodies were conjugated onto protein G beads as bait proteins, respectively. The enrichment scores of HA and NS1 sequences after one round of selection were measured by real-time PCR and normalized to input (N=3). Data are presented as mean values +/- SD.

Figure S4

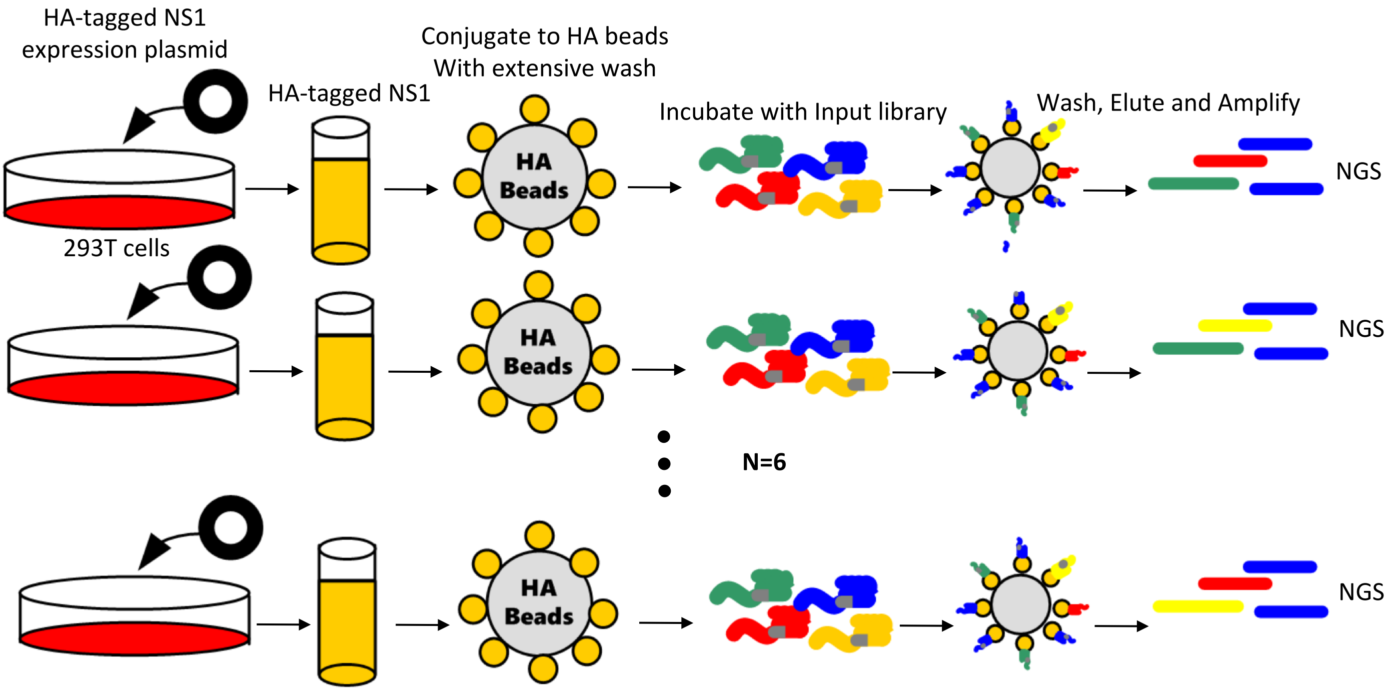

Supplementary Figure 4: Identification of cellular binders of influenza virus NS1 protein
Schematic plot shows the experimental procedures of using md-LED to identify cellular binders of NS1 protein. Briefly, NS1 with a C-terminal HA tag was expressed in 293T cells. GFP-HA were expressed as control. Then cell was lysed, centrifuged and proteins in the supernatant were conjugated to anti-HA beads at 4 degrees overnight. Five washes were performed to clean the conjugated bait proteins. These purified baits were then incubated with the input fusion libraries for three hours, precipitated, washed, and the precipitated fractions prepared for next generation sequencing as output library. A total of 6 replicates were performed.

Figure S5

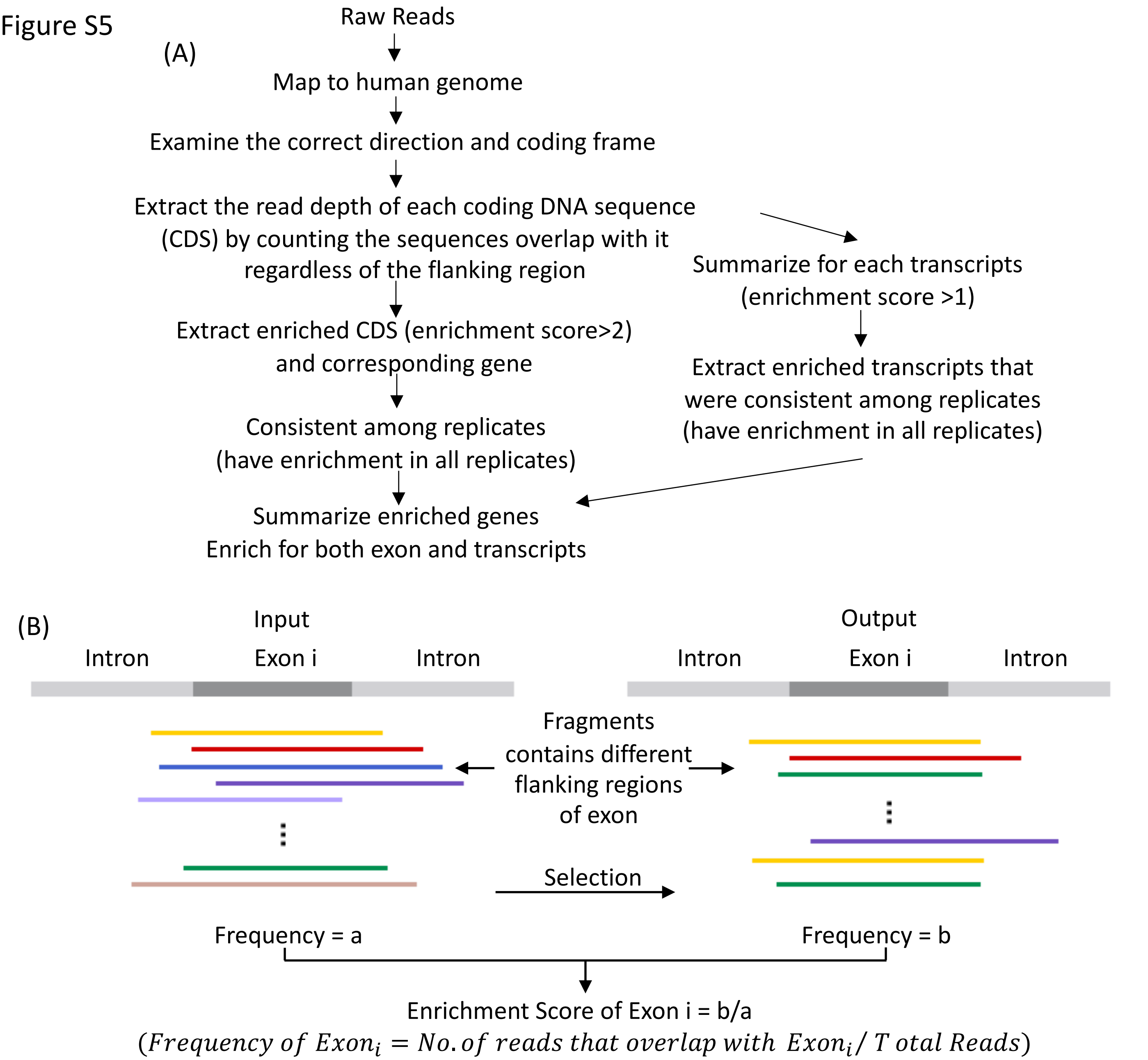

Supplementary Figure 5: Data analysis procedures

(A)The schematic diagram shows the general process of data analysis. Raw sequencing reads were mapped onto the human hg19 reference genome. Enrichment scores of each coding DNA sequence (CDS, represent the exons that encode proteins) were calculated as the relative frequency of the CDS in the selection library to that in the input library. Sequencing reads with wrong orientations or shifted reading frames were filtered out. Genes with enriched CDSs were compared between biological replicates and the consistent ones were selected. To further reduce false positive rates, we calculated the enrichment score of each gene transcript and overlapped with the enriched CDS. The enrichment score of each gene is calculated as the highest score among all gene transcripts of this gene.

(B)A schematic diagram showed the how we calculate the enrichment score of each exon. As we performed random fragmentation of the genome, each exon would be covered by many different fragments. Each fragment would contain different flanking region N- and/or C- terminus, but with the same exon region. We count the frequency of each exon as the number of sequences reads that overlap with the exon, regardless of its flanking region. And the enrichment calculated as the relative frequency of the exon in the output library (library post selection) to that in the input library.

Figure S6

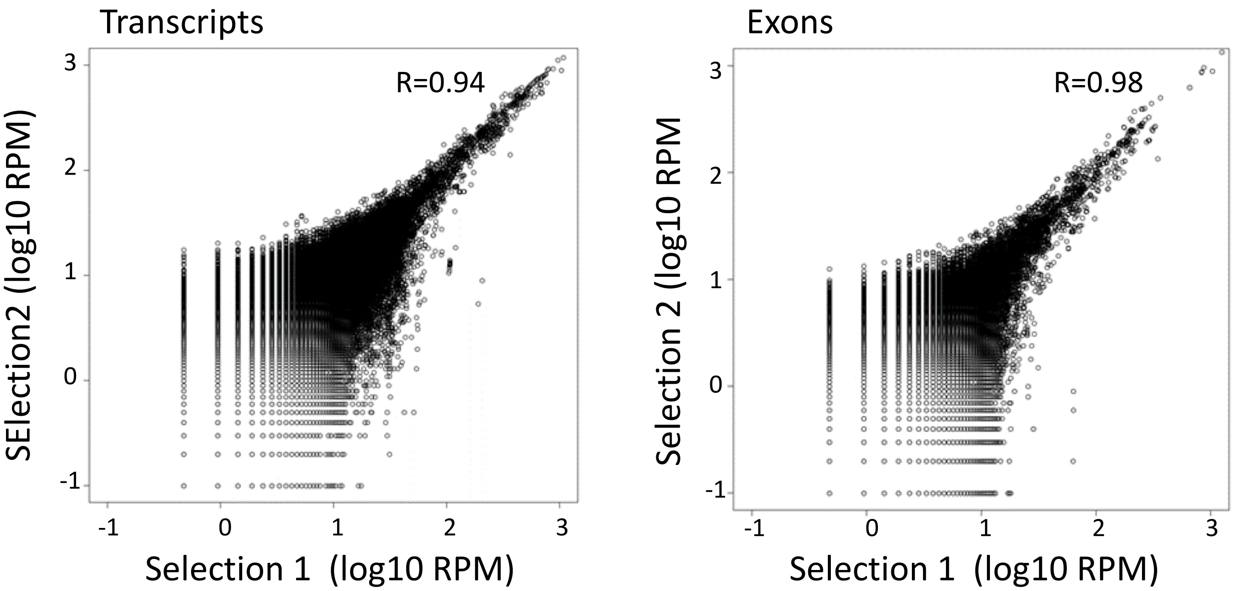

Supplementary Figure 6: Quality control of NS1 enrichment of exon library

The scatter plot shows the correlation of the frequency of gene transcripts (left panel) and exons (right panel) between two replicates, post selection against NS1 protein. The two replicates were randomly selected from the total 6 replicates that we performed, for the purpose of easier visualization. Selecting different replicates shows similar correlations (R=0.94-0.99 for pair-wise spearman correlation).

Figure S7

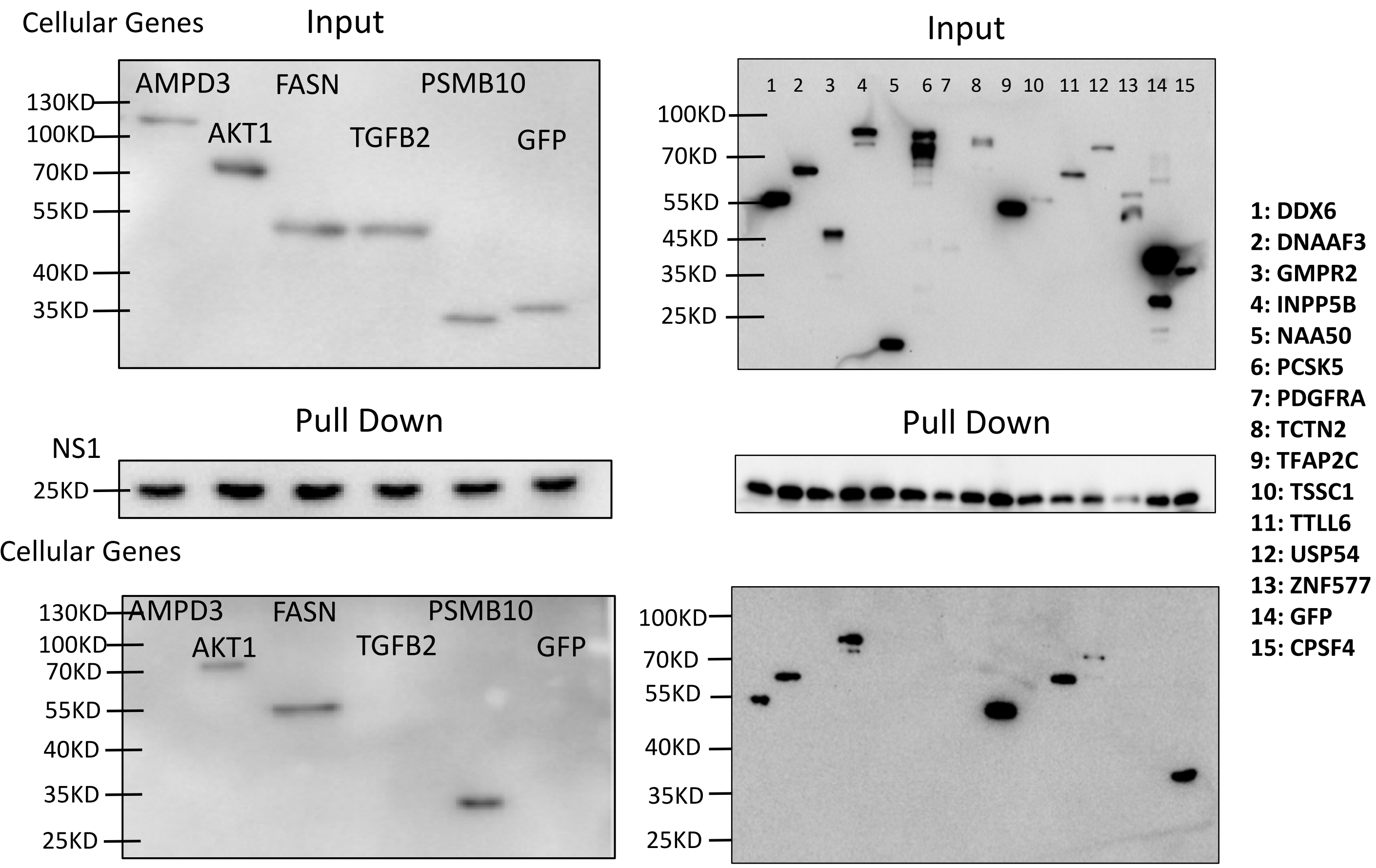

Supplementary Figure 7: Confirmation of NS1 binding cellular proteins

Complementary to Figure 2B, Interactions between NS1 proteins and the newly identified cellular binders were examined by Co-IP-western. Strep-tagged NS1 protein and FLAG-tagged cellular protein were co-expressed in 293T cells. Cells were lysed 48 h post transfection. NS1 were pull-down with HA beads and detected for co-eluted cellular proteins using FLAG antibody. 3 biological replicates were performed. Represented figures are shown.

Figure S8

GO pathways	AP-MS ($log_{10}P$)	Expanded md-LED ($log_{10}P$)
mRNA processing	-28.0	-96.7
RNA localization	-19.3	-51.3
regulation of mRNA processing	-16.0	-96.8
viral process	-11.5	0.0
ribonucleoprotein complex subunit organization	-11.0	-49.1
mRNA 3'-end processing	-10.2	-64.6
translational initiation	-10.0	-3.9
ribosome biogenesis	-8.9	0.0
mRNA surveillance pathway	-7.8	-31.5
regulation of cellular response to stress	-7.1	-1.1
RNA transport	-4.2	-96.7
regulation of mRNA metabolic process	-1.8	-49.7
mRNA spliciing	0.0	-96.7

Supplementary Figure 8: Enriched GO pathways correlated between Expanded md-LED and AP-MS
GO enrichment analysis were performed for proteins identified by expanded md-LED method and AP-MS. The enrichment score for each pathway is shown. Metascape was applied for this analysis, which utilized the hypergeometric test and Benjamini-Hochberg p-value correction algorithm.

Figure S9

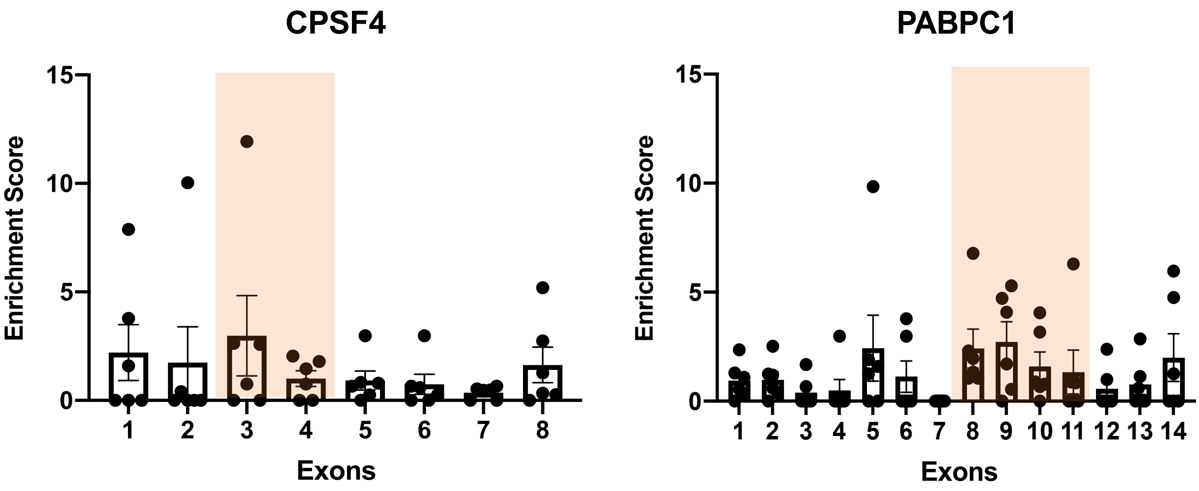

Supplementary Figure 9: md-LED facilitates the identification of binding domains.
Enrichment scores of each CDS are shown for CPSF4 (left panel) and PABPC1 (right panel). Orange shades indicates the previously reported domain interacting with NS1 (N=6). Data are presented as mean values +/- SEM.

Figure S10

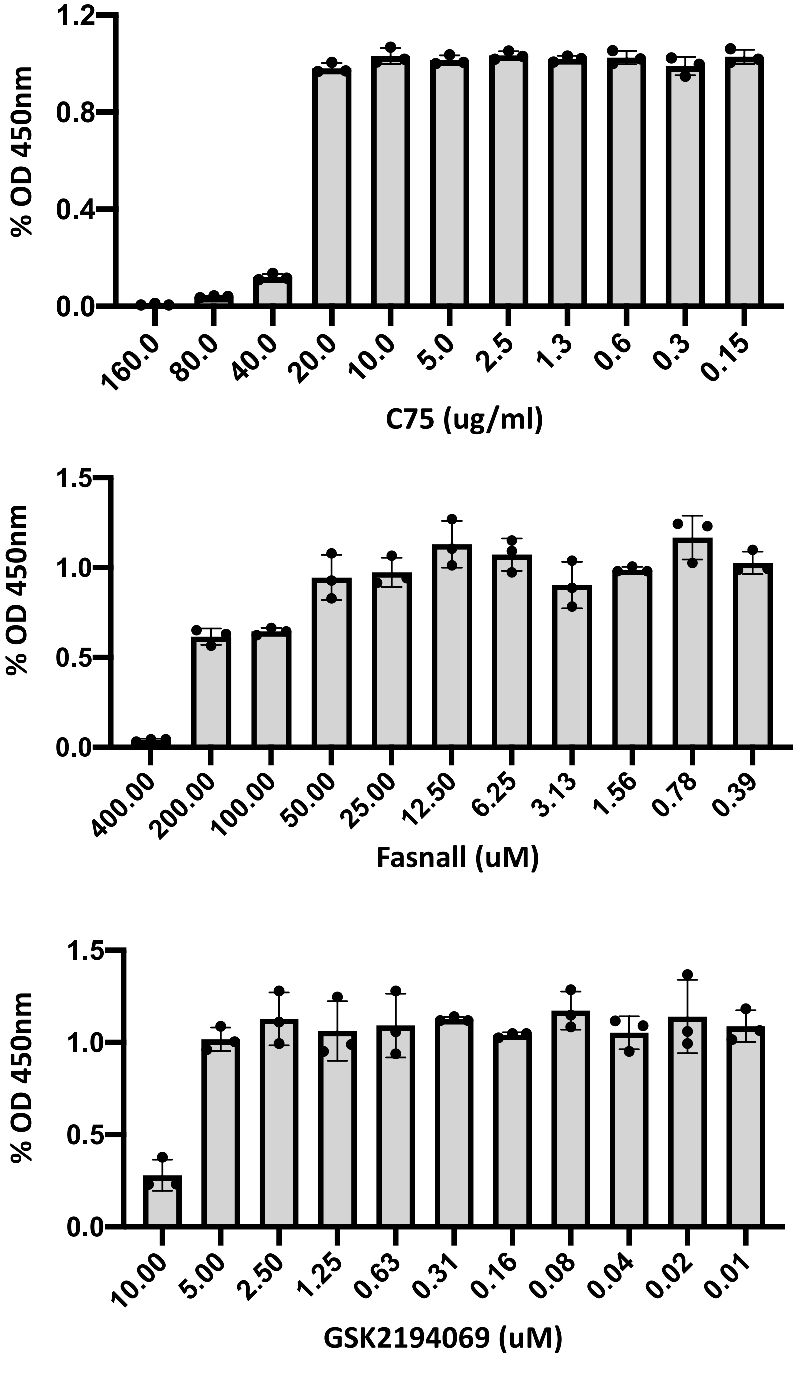

Supplementary Figure 10: Cell viability post FASN inhibitor treatment

A549 cell viability post FASN inhibitors treatment was measured by CCK8 assay at 24 hours post-treatment. The doses that we used to determine the impact FASN on viral replication have low cell toxicity (Figure 4D, N=3). Data are presented as mean values +/- SD.

Figure S11

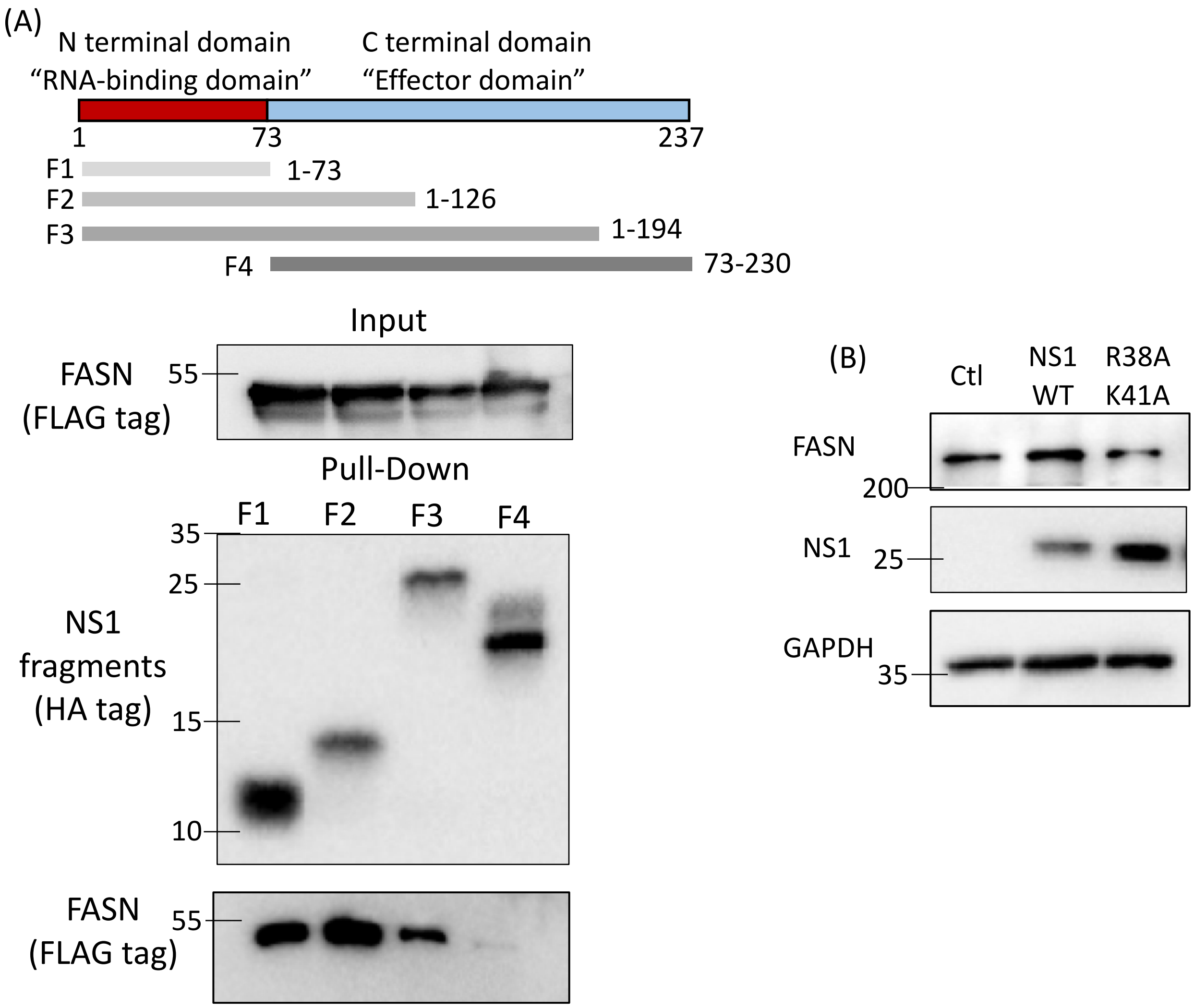

Supplementary Figure 11: N terminal domain of NS1 interact with FASN

(A) Interactions between FASN proteins and NS1 fragments were examined by immunoprecipitation (IP)-western. (B) The protein expression level of FASN was examined post WT or mutant (R38A/K41A) NS1protein over-expression. 293T cells were transfected with indicated plasmid for 48h. Endogenous FASN protein was detected with western blotting. For both panels, 3 biological replicates were performed. Represented figures are shown.

Figure S12

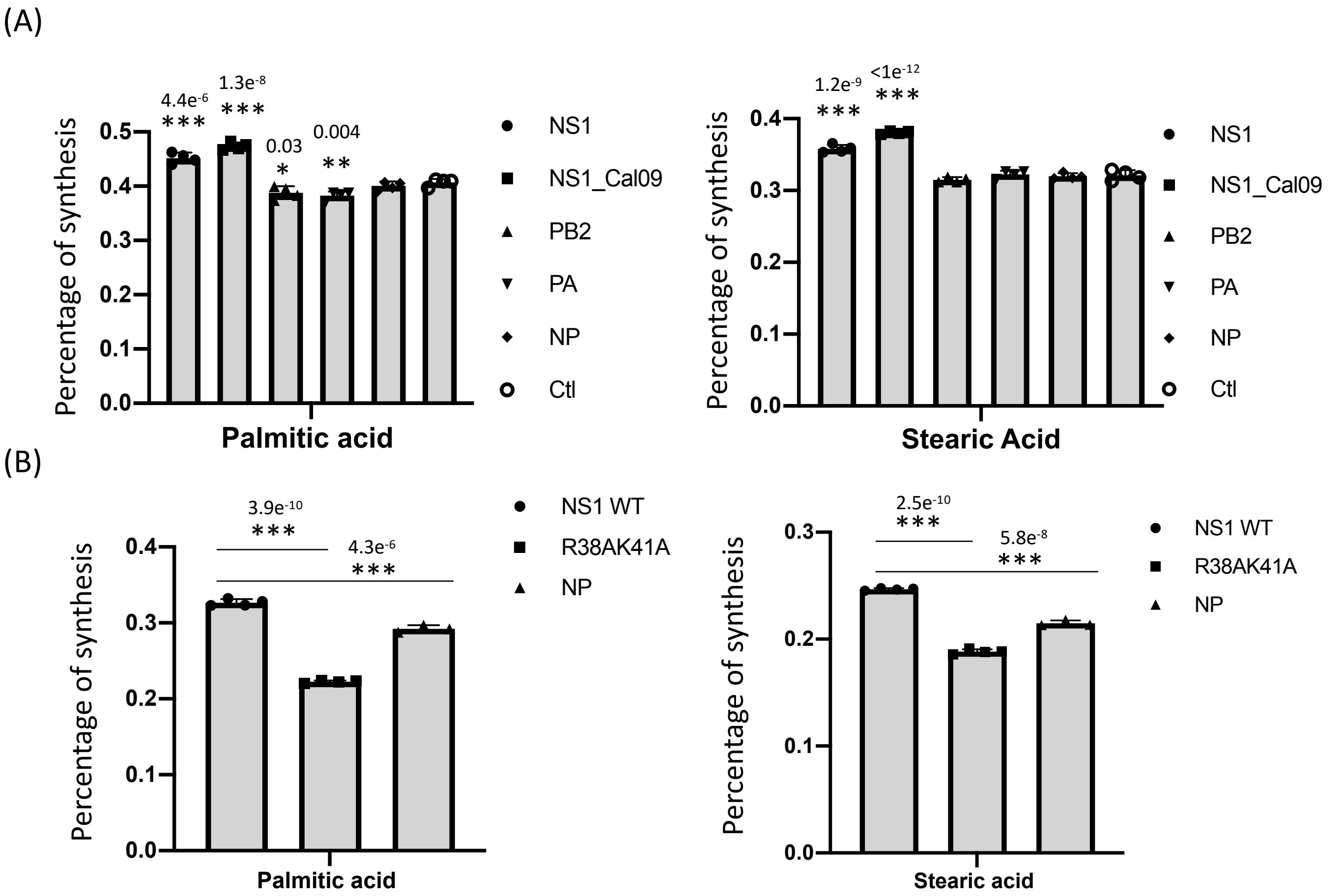

Supplementary Figure 12: Effect of WT and mutant NS1 on lipid synthesis

(A) The levels of newly synthesized fatty acid upon expression of indicated viral proteins were examined by GC/MS. The percentage of synthesized lipid over total lipid is shown for myristic acid (14:0), palmitic acid (16:0), and stearic acid (18:0). (B) The percentage of synthesized lipid over total lipid is shown for palmitic acid (16:0) and stearic acid (18:0) for WT and mutant NS1, as well as NP over-expressed A549 cells. Palmitic acid (16:0) and stearic acid (18:0) are the major product of FASN (N=4 for A and N=3-4 for B). Data are presented as mean values +/- SD. *P<0.05, **P<0.01, ***P<0.001 (one-way analysis of variance (ANOVA) with Bonferroni multiple-comparisons test for panel A, two-tailed T test for panel B).

Figure S13

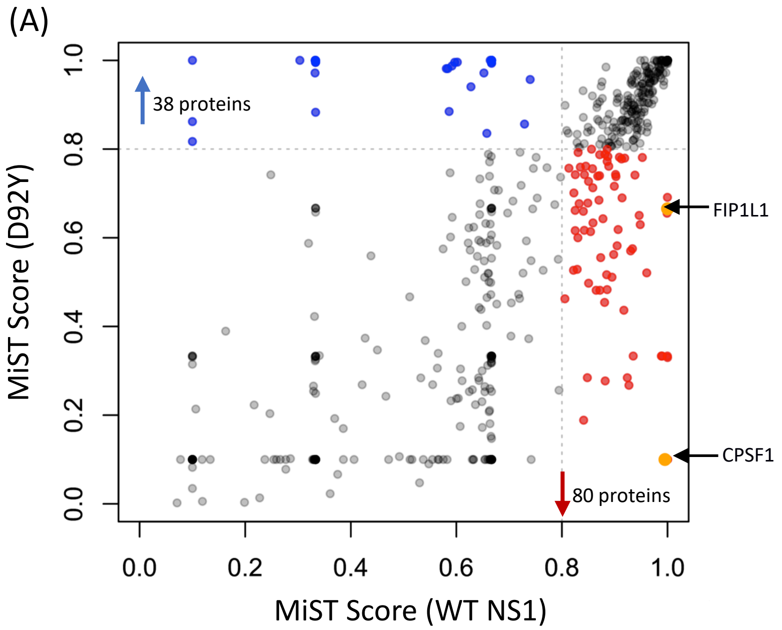

Supplementary Figure 13: D92Y NS1 mutant has reduced binding to CPSF1

(A) Scatter plot shows the MiST score of all proteins detected by AP-MS using WT or mutant NS1 protein as bait. 38 proteins showed to have increased binding to mutant D92Y protein, while 80 has reduced binding to the mutant, including CPSF1 and the known CPSF complex member FIP1L1. (B) Interactions between NS1 proteins (WT and D92Y mutant) with CPSF1 were examined by in vitro binding. FLAG-tagged CPSF1 protein were expressed in 293T cells, purified by FLAG antibody and eluted with FLAG peptides. Binding was performed by incubating purified CPSF1 with HA-tagged NS1 that conjugated to beads by HA antibody. 3 biological replicates were performed. Represented figures are shown.

Figure S14

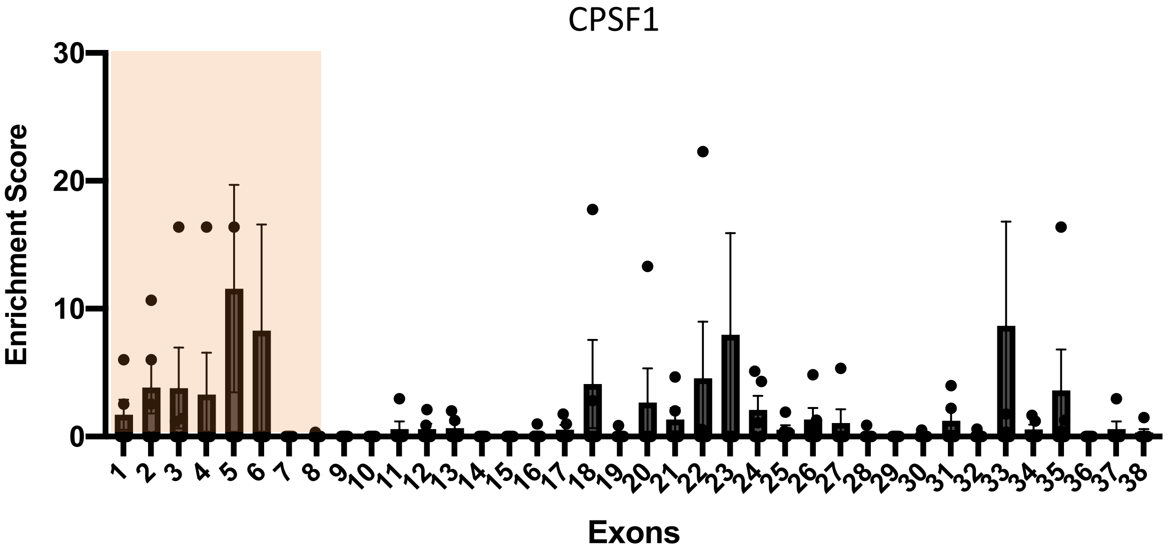

Supplementary Figure 14: Enrichment scores of CPSF1 exons.

Enrichment scores of each exon are shown for CPSF1 (N=5). Orange shades indicates the fragments interacting with NS1. Data are presented as mean values +/- SEM.

Supplementary Table 1: Primers used in current study

Name	Sequence
pF30P linker	Phospho-AAAAAAAAAAAAAAAAAAAAA-spacer9-spacer9-spacer9-ACC-puromycin
Splint	TTTTTTTTTTTTGGAGCCGCTACCCTTATCGT
T7-Rec	GGGACAATTACTATTTACAATTACCACCATGG
Lib Rev	GGAGCCGCTACCCTTATCGTCG
GFP-F	GACAACCACTACCTGAGCAC
GFP-R	GTCCATGCCGAGAGTGATC
ACTB-F	CACCCACACTGTGCCCATCTAC
ACTB-R	GTGAGGATCTTCATGAGGTAGTC
GAPDH	TGCACCACCAACTGCTTAGC
GAPDH	GGCATGGACTGTGGTCATGAG
WSN-NS1-F	CTTCGCCGAGATCAGAAG
WSN-NS1-R	CAAGAGTCATGTCAGTTA
WSN-NP-F	GAC GAT GCA ACG GCT GGT CTG
WSN-NP-R	ACC ATT GTT CCA ACT CCT TT
