# Supplementary material for: mRNA display with library of even-distribution reveals cellular interactors of influenza virus NS1
Source: Nat Commun. 2020 May 15;11:2449. doi: 10.1038/s41467-020-16140-9 (PMC7229031; doi:10.1038/s41467-020-16140-9)
Supplement: Supplementary file 3 — Reporting Summary [file 41467_2020_16140_MOESM3_ESM.pdf]

nature research

Corresponding author(s): Yushen Du; Ren Sun

Last updated by author(s): Apr 3, 2020

Reporting Summary

Nature Research wishes to improve the reproducibility of the work that we publish. This form provides structure for consistency and transparency in reporting. For further information on Nature Research policies, seeAuthors & Referees and theEditorial Policy Checklist .

Statistics

For all statistical analyses, confirm that the following items are present in the figure legend, table legend, main text, or Methods section.

n/a	Confirmed	
☐	☒	The exact sample size (*n*) for each experimental group/condition, given as a discrete number and unit of measurement
☐	☒	A statement on whether measurements were taken from distinct samples or whether the same sample was measured repeatedly
☐	☒	The statistical test(s) used AND whether they are one- or two-sided<br>*Only common tests should be described solely by name; describe more complex techniques in the Methods section.*
☒	☐	A description of all covariates tested
☒	☐	A description of any assumptions or corrections, such as tests of normality and adjustment for multiple comparisons
☐	☒	A full description of the statistical parameters including central tendency (e.g. means) or other basic estimates (e.g. regression coefficient) AND variation (e.g. standard deviation) or associated estimates of uncertainty (e.g. confidence intervals)
☐	☒	For null hypothesis testing, the test statistic (e.g. *F*, *t*, *r*) with confidence intervals, effect sizes, degrees of freedom and *P* value noted<br>*Give P values as exact values whenever suitable.*
☒	☐	For Bayesian analysis, information on the choice of priors and Markov chain Monte Carlo settings
☒	☐	For hierarchical and complex designs, identification of the appropriate level for tests and full reporting of outcomes
☒	☐	Estimates of effect sizes (e.g. Cohen's *d*, Pearson's *r*), indicating how they were calculated

Our web collection on statistics for biologists contains articles on many of the points above.

Software and code

Policy information about availability of computer code

Data collection	Cutadapt (version 2.1) were used to trim off adaptor sequence, Bowtie2 was used for mapping, bedtools (v2.17.0) were used to intersect mapped reads with exons. Custom python scripts for further data analysis were deposited to github : https://github.com/YushenDu/PED-
Data analysis	https://github.com/YushenDu/PED-

For manuscripts utilizing custom algorithms or software that are central to the research but not yet described in published literature, software must be made available to editors/reviewers. We strongly encourage code deposition in a community repository (e.g. GitHub). See the Nature Research guidelines for submitting code & software for further information.

Data

Policy information about availability of data

All manuscripts must include a data availability statement. This statement should provide the following information, where applicable:

- Accession codes, unique identifiers, or web links for publicly available datasets
- A list of figures that have associated raw data
- A description of any restrictions on data availability

Scored mass spectrometry data files have been uploaded here as Table S2.The Raw data of Mass-spectrometry can be found in: https://www.dropbox.com/sh/0gesb457b70t3tr/AACkdHEgILH-H94xgQyKT-r7a?dl=0. The sequencing data are deposited to NIH Short Read Archive (SRA) with access numbers PRJNA520773 (https://www.ncbi.nlm.nih.gov/bioproject/520773) and PRJNA383938 (ncbi.nlm.nih.gov/bioproject/?term=PRJNA383938). The mass spectrometry proteomics data of current study have been deposited to the ProteomeXchange Consortium via the PRIDE partner repository with the dataset identifier PXD018377. Source data for Fig. 2A, Fig. 3B, 3C and 3D have uploaded here as Supplementary Table 1 and 3. Source data for Fig. 2B, Fig. 3D, Fig 4 A-H, Fig. 5B, 5D, 5E, 5F were uploaded as Source Data file. Databases used in this study also includes: STRING database, CORUM database, VirHost database, PRIDE (project: PXD000418), PDB: 2RHK.

Field-specific reporting

Please select the one below that is the best fit for your research. If you are not sure, read the appropriate sections before making your selection.

- [x] Life sciences
- [ ] Behavioural & social sciences
- [ ] Ecological, evolutionary & environmental sciences

For a reference copy of the document with all sections, see nature.com/documents/nr-reporting-summary-flat.pdf

Life sciences study design

All studies must disclose on these points even when the disclosure is negative.

Sample size	Experiments were performed with at least three biological replicates. For NS1 PPI screening using md-LED, six biological replicates were performed. For AP-MS, three biological replicates with and without IFN selection were performed.
Data exclusions	No data were excluded.
Replication	Biological experiments were performed with at least three biological replicates with consistent results. Screening results from all replicates were shown in the paper.
Randomization	Samples were randomly allocated.
Blinding	Group allocation were blinded to investigators when possible.

Reporting for specific materials, systems and methods

We require information from authors about some types of materials, experimental systems and methods used in many studies. Here, indicate whether each material, system or method listed is relevant to your study. If you are not sure if a list item applies to your research, read the appropriate section before selecting a response.

Materials & experimental systems

n/a	Involved in the study	
☐	☒	Antibodies
☐	☒	Eukaryotic cell lines
☒	☐	Palaeontology
☒	☐	Animals and other organisms
☒	☐	Human research participants
☒	☐	Clinical data

Methods

n/a	Involved in the study	
☒	☐	ChIP-seq
☒	☐	Flow cytometry
☒	☐	MRI-based neuroimaging

Antibodies

Antibodies used

DYKDDDDK Tag Monoclonal Antibody (FG4R, ThermoFisher, Catalog # MA1-91878); Monoclonal Anti-Strep Tag antibody (Sigma-Aldrich, Cat.SAB2702216-100UL); Strep-Tactin®-HRP conjugate (IBA, Cat. 2-1502-001); DYKDDDDK Tag Monoclonal Antibody (FG4R), HRP (ThermorFisher, Catalog # MA1-91878-HRP); HA Tag Monoclonal Antibody (2-2.2.14) (ThermoFisher, Catalog # 26183), Polyclonal Influenza A virus NS1 (nonstructural protein) antibody (GeneTex, Cat No. GTX125990); Monoclonal Influenza A virus NS1 (nonstructural protein) antibody [GT1653] (GeneTex, Cat No. GTX633685). Fatty Acid Synthase (C20G5) Rabbit mAb #3180 (CST, Cat. # 3180S); Monoclonal Anti-GAPDH antibody (Sigma,Cat: G8795-200UL); beta Tubulin Monoclonal Antibody (ThermoFisher, Catalog # 32-2600); M2 anti-Flag beads (Sigma-Aldrich, Cat. A2220) ; Strep-Tactin Sepharose beads (IBA Lifesciences, Cat: 2-1201-010) ; EZview™ Red Anti-HA Affinity Gel (Sigma, Cat. E6779-1ML)

Validation

DYKDDDDK Tag Monoclonal Antibody (FG4R, ThermoFisher, Catalog # MA1-91878):
Species: Amphibian, Human, Tag
Applications:ICC, IF, IP, WB, ELISA, IHC, Array
https://www.thermofisher.com/antibody/product/DYKDDDDK-Tag-Antibody-clone-FG4R-Monoclonal/MA1-91878

Monoclonal Anti-Strep Tag antibody (Sigma-Aldrich, Cat.SAB2702216-100UL);
Applications ICC/IF, WB
https://www.sigmaaldrich.com/catalog/product/sigma/sab2702215?lang=en&region=US

HA Tag Monoclonal Antibody (2-2.2.14) (ThermoFisher, Catalog # 26183),
Species: Human, Tag
Applications:ICC, IF, IP, WB, IHC, ChIP, PLA
https://www.thermofisher.com/antibody/product/HA-Tag-Antibody-clone-2-2-2-14-Monoclonal/26183

Polyclonal Influenza A virus NS1 (nonstructural protein) antibody (GeneTex, Cat No. GTX125990);
Applications:WB, ICC/IF, IHC-P
https://www.genetex.com/Product/Detail/Influenza-A-virus-NS1-nonstructural-protein-antibody/GTX125990

Monoclonal Influenza A virus NS1 (nonstructural protein) antibody [GT1653] (GeneTex, Cat No. GTX633685).
Applications: WB
https://www.genetex.com/Product/Detail/Influenza-A-virus-NS1-nonstructural-protein-antibody-GT1653/GTX633685

Fatty Acid Synthase (C20G5) Rabbit mAb #3180 (CST, Cat. # 3180S);
Applications: WB, IP, IHC, IF
Reactivity: H, M, R
https://www.cellsignal.com/products/primary-antibodies/fatty-acid-synthase-c20g5-rabbit-mab/3180

Monoclonal Anti-GAPDH antibody (Sigma,Cat: G8795-200UL);
Species: mouse, mink, rabbit, rat, human, hamster, canine, turkey, chicken, monkey, bovine
Applications: WB, IHC, ELISA
https://www.sigmaaldrich.com/catalog/product/sigma/g8795?lang=en&region=US

beta Tubulin Monoclonal Antibody (ThermoFisher, Catalog # 32-2600);
Species: Dog, C. elegans, Human, Mouse, Non-human primate, Rat
Applications:ICC, IF, IP, WB, Flow, IHC
https://www.thermofisher.com/antibody/product/beta-Tubulin-Antibody-clone-2-28-33-Monoclonal/32-2600

Eukaryotic cell lines

Policy information about cell lines

Cell line source(s)	293T and A549 cells were thawed from Dr. Ren Sun's storage. 293T originally was bought from ADCC, A549 was a gift from Dr. Steve Dubinett's lab in UCLA.
Authentication	Dr. Steven Dubinett lab perform A549 cell line verification (finger printing) regularly. 293T cells was not authenticated regularly.
Mycoplasma contamination	Cell lines were confirmed with no mycoplasma contaminiation.
Commonly misidentified lines (See ICLAC register)	No misidentified lines were used according to ICLAC version 9.
