# Supplementary material for: mRNA display with library of even-distribution reveals cellular interactors of influenza virus NS1
Source: Nat Commun. 2020 May 15;11:2449. doi: 10.1038/s41467-020-16140-9 (PMC7229031; doi:10.1038/s41467-020-16140-9)
Supplement: Supplementary file 4 — Description of Additional Supplementary Files [file 41467_2020_16140_MOESM4_ESM.pdf]

Description of Additional Supplementary Files

File Name: Supplementary Data 1
Description: 25 proteins were identified by md-LED as potential interactors with NS1. The enrichment score is shown for individual protein. The confirmed NS1 binders by Co-IP is marked.

File Name: Supplementary Data 2
Description: The raw MiST score outputs are shown for the AP-MS experiments using WT and mutant (D92Y) NS1 as bait.

File Name: Supplementary Data 3
Description: Potential NS1 interactors identified by md-LED, expanded md-LED, AP-MS and the ones reported in literatures are listed.
